# Clinical outcome of drug-coated balloons in patients with femoropopliteal chronic total occlusive lesions: results from the multicenter EAGLE study

**DOI:** 10.1186/s42155-022-00329-8

**Published:** 2022-10-06

**Authors:** Naoki Hayakawa, Mitsuyoshi Takahara, Tatsuya Nakama, Kazunori Horie, Keisuke Takanashi, Teruaki Kanagami, Shinya Ichihara, Masataka Arakawa, Kazuki Tobita, Shinsuke Mori, Yo Iwata, Kenji Suzuki, Junji Kanda

**Affiliations:** 1 Department of Cardiovascular Medicine, Asahi General Hospital, I-1326 Asahi, Chiba, 289-2511 Japan; 2 Department of Metabolic Medicine, Osaka University Graduate School of Medicine, Osaka, Japan; 3 Department of Cardiology, Tokyo Bay Medical Center, Urayasu, Japan; 4 Division of Vascular Surgery, Department of Surgery, Jikei University School of Medicine, Tokyo, Japan; 5 Department of Cardiovascular Medicine, Sendai Kousei Hospital, Sendai, Japan; 6 Department of Cardiology, Shonan Kamakura General Hospital, Kamakura, Japan; 7 Department of Cardiology, Saiseikai Yokohama City Eastern Hospital, Yokohama, Japan; 8 Department of Cardiology, Funabashi Municipal Medical Center, Funabashi, Japan; 9 Department of Cardiology, Tokyo Saiseikai Central Hospital, Tokyo, Japan

**Keywords:** Drug-coated balloon, Chronic total occlusive lesion, Endovascular therapy, Femoropopliteal occlusive disease

## Abstract

**Background:**

Several studies have reported the efficacy of drug-coated balloons (DCB) for simple femoropopliteal (FP) lesions. However, the effectiveness of DCB for FP chronic total occlusive lesions (CTO) is controversial. The present study investigated the clinical outcomes of DCB for FP-CTO.

**Materials and methods:**

We retrospectively analyzed 359 limbs of 318 patients who underwent endovascular therapy with DCB for FP-CTO between July 2017 and February 2021 at seven cardiovascular centers. The primary endpoint was 12-month primary patency. The secondary endpoints were the 12-month rates of freedom from: (1) clinically-driven target lesion revascularization (CD-TLR), and (2) re-occlusion. The association of baseline characteristics with the 12-month restenosis risk was investigated using the Cox proportional hazards regression model.

**Results:**

The 12-month rate of primary patency was 79.8% (95% confidence interval [95%CI], 75.1% to 84.8%), whereas the corresponding rates of freedom from CD-TLR and re-occlusion were 86.4% (95%CI: 82.6% to 90.4%) and 88.5% (95%CI: 84.7% to 92.4%), respectively. The bailout stent rate was 8.9%. Independent risk factors for restenosis were hemodialysis (adjusted hazard ratio, 2.18 [1.39 to 3.45]; *P* = 0.001), chronic limb-threatening ischemia (CLTI) (2.02 [1.33 to 3.07]; *P* = 0.001), and restenosis lesion (2.02 [1.32 to 3.08]; *P* = 0.001). Use of dual antiplatelet therapy (DAPT) was identified as a protective factor for restenosis (0.54 [0.35 to 0.82]; *P* = 0.003).

**Conclusions:**

Despite the low rate of bailout stent, DCB treatment for FP-CTO was effective in real-world clinical practice. Hemodialysis, CLTI, and restenosis lesion were independent risk factors for 12-month restenosis, and the use of DAPT significantly attenuated the risk of 12-month restenosis.

## Introduction

Currently, the prevalence of peripheral artery disease (PAD) has dramatically increased in aging societies (Song et al. 2019). Endovascular therapy (EVT) is considered a first-line strategy for femoropopliteal (FP) occlusive disease because of its low invasiveness, fewer complications, and improved durability related to advances in devices and current techniques (Aboyans et al. 2018; Bailey et al. 2019). Favorable clinical results of drug-coated balloons (DCB) have been reported for simple FP lesions (Iida et al. 2018; Laird et al. 2019; Tepe et al. 2015). However, for complex FP lesions, its use is still controversial. In particular, for chronic total occlusive lesion (CTO), the most challenging situation regarding FP lesions, the clinical benefit of DCB has been variable; unfavorable and outstanding results have been reported (AbuRahma et al. 2019; Hayakawa et al. 2022a, 2022b). Previous studies of DCB treatment for FP-CTO or FP-complex lesions (including a high percentage of CTO) have reported an acceptable 12-month primary patency (78% to 85%) (Tepe et al. 2019; Liistro et al. 2019). However, in these studies, 20%–50% of cases required bailout stents indicating the findings were not related to the “pure” effect of DCB itself. The actual performance of DCB on complex FP lesions, especially on CTO, is unknown. Therefore, in the present study, we investigated the clinical outcomes of DCB for FP-CTO. We avoided using bailout stents after DCB where possible to utilize the advantage of the “leaving nothing behind” strategy.

## Materials and methods

### Study population and design

The EAGLE (Clinical result of **E**ndov**A**scular treatment with dru**G**-coated ba**L**loon for f**E**moropopliteal chronic total occlusion) study is a multicenter, retrospective analysis from the prospectively maintained database. Between April 2017 and February 2021, 3635 consecutive symptomatic PAD patients with FP lesions received EVT, and 1457 lesions had CTO segments. Of these, 359 lesions (318 patients) were treated with DCB and enrolled in this study. Patients with non-atherosclerotic disease including vasculitis or systemic inflammatory disease, life expectancy of less than 1 year, advanced malignancy, acute limb ischemia, and congenital anatomical abnormalities such as persistent sciatic artery, or aneurysmal lesions were excluded. The selection of DCB (high dose or low dose) was decided based on each operators’ decision.

The study protocol was approved by the local ethics committee at all participating centers, and the study was performed in accordance with the Declaration of Helsinki. The requirement for informed consent was waived because of the retrospective study design, in which existing medical records were used. Alternatively, patients could opt out of the study. Relevant information regarding the study is available to the public in accordance with the Ethical Guidelines for Medical and Health Research Involving Human Subjects.

### Procedural protocol

Aspirin, clopidogrel, prasugrel, cilostazol, or oral anticoagulant drugs were started at least 24 hours before the procedure. Dual antiplatelet therapy (DAPT), defined as the administration of aspirin and clopidogrel or prasugrel, was used at least 1 month after EVT. After the insertion of a guiding sheath from the ipsilateral or contralateral femoral artery, 0.014-, 0.018-, or 0.035-in. guidewires were used with a back-up support catheter. A bi-directional approach was conducted as needed. The type of pre-dilation balloon used (semi-compliant, non-compliant, cutting, or scoring balloon) depended on the operator. DCB was used after confirming as much as possible that the residual stenosis was < 50% and the degree of dissection was less than grade D (Fujihara et al. 2017; Feldman et al. 2018). The evaluation of a pressure gradient was performed as required, and pressure gradients < 10 mmHg were defined as significant stenosis (Tepe et al. 2015). After successful lesion preparation, the target lesion was fully covered by the DCB (geographic mismatches were carefully avoided). If > 50% residual stenosis or The National Heart, Lung and Blood Institute (NHLBI) grade D or higher dissection was observed after using DCB, bailout stenting was considered (the necessity of stent use was finally judged by each operator). Atherectomy devices were not used in this study because they were not commercially available in our country during the study period. Procedures and measurements were performed in each institute by at least two or more specialists from the Japanese Association of Cardiovascular Intervention and Therapeutics.

### Study endpoints and definitions

Procedural success was defined as < 50% residual stenosis without angiographic flow-limiting dissection. Calcification severity was evaluated by the Peripheral Arterial Calcium Scoring System (PACSS) grade (Rocha-Singh et al. 2014). The NHLBI and Kobayashi classifications were used to evaluate the severity of the dissection (Fujihara et al. 2017; Kobayashi et al. 2018). High-dose DCB (3.5 μg/mm^2^) was IN.PACT Admiral DCB (Medtronic Vascular, Santa Clara, CA, USA), and low-dose DCB (2.0 μg/mm^2^) was Lutonix RX DCB (Becton, Dickinson and Company, Franklin Lakes, NJ, USA) or Ranger DCB (Boston Scientific Corporation, Marlborough, MA, USA).

The primary endpoint of this study was 12-month primary patency, defined as freedom from restenosis. We defined restenosis as 1) a peak systolic velocity ratio ≥ 2.5 by duplex ultrasound (DUS), or 2) a vessel with > 50% diameter stenosis by computed tomography angiography (CTA) or angiography. The secondary endpoints were 12-month freedom from 1) clinical driven target lesion revascularization (CD-TLR) and 2) re-occlusion. The rates of major amputation and all-cause mortality were also evaluated. Patency of the target vessel was evaluated by DUS, CTA, or angiography depending on the recommendations of the Society of Vascular Surgery: every 3 months during the first year, or every 6 months or annually thereafter (Stoner et al. 2016).

### Statistical analysis

Data are presented as the mean ± standard deviation for continuous variables and as a percentage for categorical variables, unless otherwise indicated. A *P*-value < 0.05 was considered statistically significant, and 95% confidence intervals (CIs) were reported when appropriate. Time-to-events were estimated by the Kaplan-Meier method. The association of baseline characteristics with the 12-month restenosis risk was investigated using the Cox proportional hazards regression model. The variables with statistical significance in the univariate model were entered into the multivariate model. Missing data were addressed using the multiple imputation by chained equations method. In this procedure, we generated five imputed datasets and combined the analytic results based on Rubin’s rule. All statistical analyses were performed with R, version 4.1.1 (R Development Core Team, Vienna, Austria).

## Results

### Baseline characteristics

Clinical characteristics are summarized in Table 1. The mean age was 75 ± 9 years and 66.0% were male. The prevalences of diabetes mellitus and hemodialysis were 62.6% and 20.4%, respectively. Perioperative DAPT use was 66.4%, cilostazol use was 23.0%, and anticoagulant use was 17.9%. The percentages of CLTI, history of EVT, and popliteal involved lesions were 32.9%, 26.7%, and 47.6%, respectively. The reference vessel diameter was 5.1 ± 0.8 mm, the lesion length was 21.9 ± 10.0 cm, and the occlusion length was 14.5 ± 10.6 cm. PACCS grade 3 was observed in 10.0% of cases, and grade 4 in 23.1%. High-dose DCB use was 68.5% and intravascular ultrasound (IVUS) use was 77.2%. The rate of bailout stent was 8.9%.

**Table 1:** Baseline characteristics

Patient characteristics	(*n* = 318)	
Male sex	210 (66.0%)	
Age (years)	75 ± 9	
Diabetes mellitus	199 (62.6%)	
Smoking	76 (23.9%)	
Renal failure on dialysis	65 (20.4%)	
Cerebrovascular disease	82 (25.8%)	
Coronary artery disease	151 (47.5%)	
Chronic heart failure	68 (21.4%)	
Dual antiplatelet therapy	211 (66.4%)	
Cilostazol use	73 (23.0%)	
Anticoagulant use	57 (17.9%)	
β-blocker use	120 (37.7%)	
Renin-angiotensin system inhibitor use	169 (53.1%)	
Statin use	207 (65.1%)	
Limb characteristics	(*n* = 359)	
Chronic limb-threatening ischemia	118 (32.9%)	
Ankle brachial index	0.60 ± 0.16	(missing data, *n* = 64)
Restenosis lesion	96 (26.7%)	
Lesion location		
SFA	142 (39.6%)	
SFA-Pop A	155 (43.2%)	
SFA-BTK	12 (3.3%)	
Pop A	33 (9.2%)	
Pop A-BTK	17 (4.7%)	
Popliteal involvement	217 (60.4%)	
Reference vessel diameter (mm)	5.1 ± 0.8	
Lesion length (mm)	218.8 ± 100.0	
Length of chronic total occlusion (mm)	145.3 ± 106.4	
PACSS classification		
Grade 0	173 (48.2%)	
Grade 1	48 (13.4%)	
Grade 2	19 (5.3%)	
Grade 3	36 (10.0%)	
Grade 4	83 (23.1%)	
Below-the-knee runoff		(missing data, *n* = 1)
No runoff	16 (4.5%)	
1 runoff	150 (41.9%)	
2 runoffs	131 (36.6%)	
3 runoffs	61 (17.0%)	
High-dose DCB use	246 (68.5%)	
Mean diameter of DCB (mm)	5.1 ± 0.7	(missing data, *n* = 38)
Total length of DCB (mm)	232.7 ± 102.1	(missing data, *n* = 38)
Intravascular ultrasound use	277 (77.2%)	
Stent implantation	32 (8.9%)	
Dissection grade NHLBI ≥ C	58 (16.2%)	
Dissection grade Kobayashi ≥1/3	46 (12.8%)	
Subintimal wire passage	60 (21.7%)	(missing data, *n* = 83)
Degree of calcification		(missing data, *n* = 81)
0°	85 (30.6%)	
1 to 90°	83 (29.9%)	
91 to 180°	29 (10.4%)	
181 to 270°	24 (8.6%)	
271 to 360°	57 (20.5%)	
Calcified nodule	58 (20.8%)	(missing data, *n* = 80)
Degree of dissection after DCB		(missing data, *n* = 155)
0°	62 (30.4%)	
1 to 90°	82 (40.2%)	
91 to 180°	52 (25.5%)	
181 to 270°	7 (3.4%)	
271 to 360°	1 (0.5%)	
Minimum lumen area (mm^2^)	12.7 ± 5.1	(missing data, *n* = 161)
Distal EEM area (mm^2^)	29.8 ± 11.8	(missing data, *n* = 92)
Distal lumen area (mm^2^)	17.0 ± 6.9	(missing data, *n* = 122)
Major amputation	4 (1.3%)	
All-cause mortality	45 (14.2%)	

### Outcome measures

**Fig. 1:**
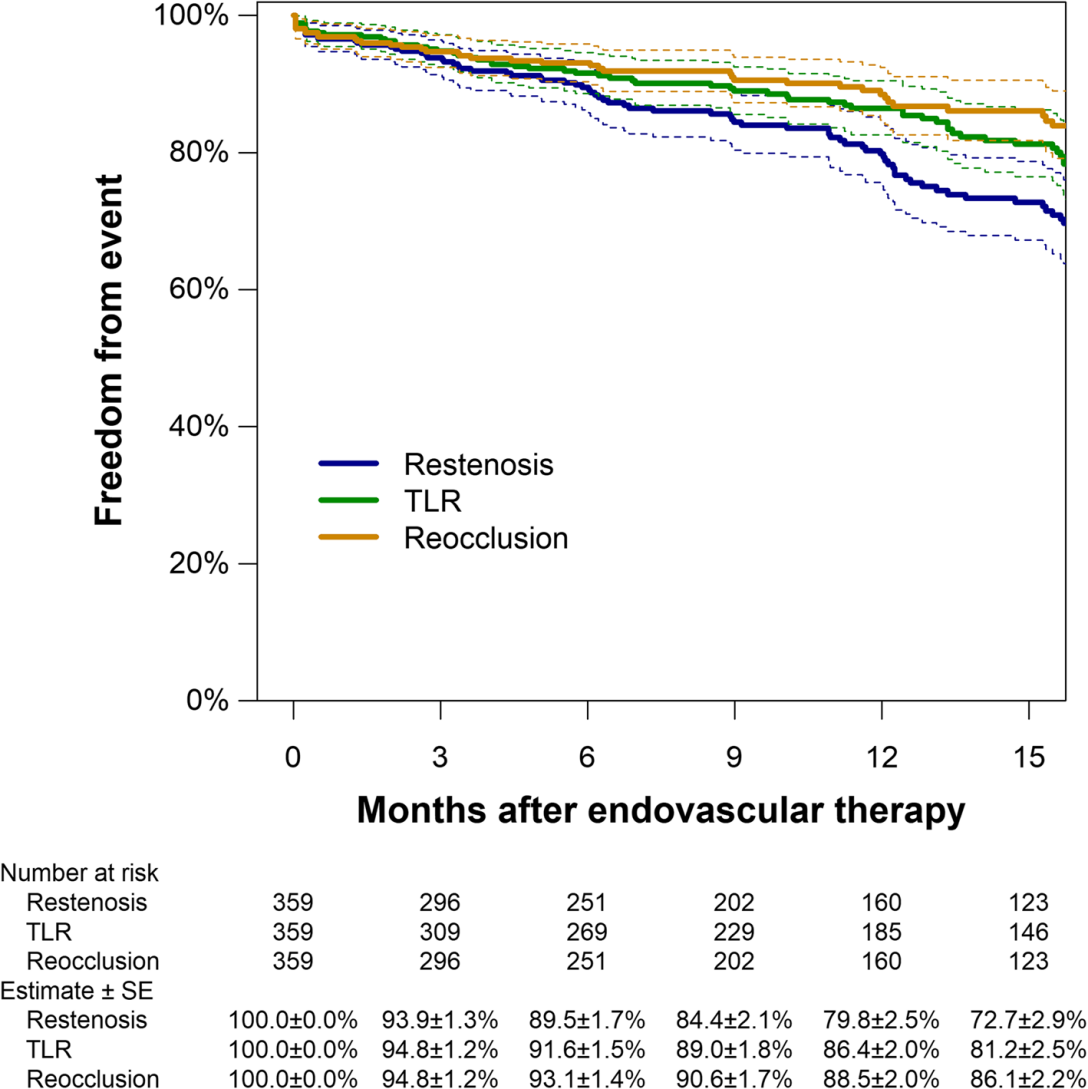
Kaplan Meier estimates of primary patency, freedom from TLR, and freedom from re-occlusion. Dotted lines indicate 95% confidence intervals. SE, standard error

The median follow-up period was 10.9 months (interquartile range: 4.8–18.3). The rate of 12-month major amputation and all-cause mortality was 1.3% and 14.2%, respectively. During the study period, restenosis was detected in 93 cases. Figure 1 illustrates the Kaplan Meier estimates of primary patency and freedom from CD-TLR. The 12-month primary patency was 79.8% (95%CI: 75.1% to 84.8%), whereas the corresponding rates of freedom from CD-TLR and re-occlusion were 86.4% (95% CI: 82.6% to 90.4%) and 88.5% (95% CI: 84.7% to 92.4%), respectively. Table 2 shows the association of the clinical characteristics with the 12-month restenosis risk. Clinical features independently associated with restenosis risk were hemodialysis (adjusted hazard ratio, 2.18 [1.39 to 3.45]; *P* = 0.001), CLTI (2.02 [1.33 to 3.07]; *P* = 0.001), and restenosis lesion (2.02 [1.32 to 3.08]; *P* = 0.001). The administration of DAPT significantly attenuated the risk of 12-month restenosis (0.54 [0.35 to 0.82]; *P* = 0.003).

**Table 2:** Association of baseline characteristics with 1-year restenosis risk

	Unadjusted hazard ratio	Adjusted hazard ratio
Male sex	0.78 [0.51 to 1.18] (*P* = 0.24)	N/I
Age (years)	0.98 [0.96 to 1.00] (*P* = 0.12)	N/I
Diabetes mellitus	1.02 [0.67 to 1.55] (*P* = 0.94)	N/I
Smoking	1.39 [0.89 to 2.20] (*P* = 0.15)	N/I
Renal failure on dialysis	2.35 [1.50 to 3.66] (*P* < 0.001)	2.18 [1.39 to 3.45] (*P* = 0.001)
Cerebrovascular disease	1.49 [0.95 to 2.32] (*P* = 0.081)	N/I
Coronary artery disease	0.78 [0.52 to 1.18] (*P* = 0.24)	N/I
Chronic heart failure	0.84 [0.50 to 1.43] (*P* = 0.52)	N/I
Dual antiplatelet therapy	0.62 [0.41 to 0.94] (*P* = 0.023)	0.54 [0.35 to 0.82] (*P* = 0.003)
Cilostazol use	1.11 [0.71 to 1.73] (*P* = 0.66)	N/I
Anticoagulant use	1.23 [0.74 to 2.03] (*P* = 0.43)	N/I
β blocker use	1.04 [0.68 to 1.59] (*P* = 0.86)	N/I
Renin-angiotensin system inhibitor use	0.82 [0.54 to 1.23] (*P* = 0.33)	N/I
Statin use	0.93 [0.61 to 1.43] (*P* = 0.75)	N/I
Chronic limb-threatening ischemia	2.19 [1.45 to 3.29] (*P* < 0.001)	2.02 [1.33 to 3.07] (*P* = 0.001)
Ankle brachial index	1.30 [0.20 to 8.53] (*P* = 0.77)	N/I
Restenosis lesion	1.98 [1.30 to 3.01] (*P* = 0.001)	2.02 [1.32 to 3.08] (*P* = 0.001)
Popliteal involvement	1.25 [0.83 to 1.88] (*P* = 0.28)	N/I
Reference vessel diameter (mm)	0.81 [0.62 to 1.06] (*P* = 0.13)	N/I
Lesion length (mm)	1.00 [1.00 to 1.00] (*P* = 0.61)	N/I
Length of chronic total occlusion (mm)	1.00 [1.00 to 1.00] (*P* = 0.082)	N/I
PACSS classification	1.05 [0.93 to 1.18] (*P* = 0.43)	N/I
Below-the-knee runoff	1.99 [0.87 to 4.56] (*P* = 0.11)	N/I
High-dose DCB use	0.71 [0.47 to 1.08] (*P* = 0.11)	N/I
Mean diameter of DCB (mm)	0.74 [0.35 to 1.55] (*P* = 0.36)	N/I
Total length of DCB (mm)	1.00 [1.00 to 1.00] (*P* = 0.99)	N/I
Intravascular ultrasound use	0.85 [0.53 to 1.36] (*P* = 0.49)	N/I
Stent implantation	1.17 [0.57 to 2.42] (*P* = 0.67)	N/I
Dissection grade NHLBI ≥ C	1.40 [0.83 to 2.37] (*P* = 0.21)	N/I
Dissection grade Kobayashi ≥1/3	1.66 [0.94 to 2.94] (*P* = 0.081)	N/I
Subintimal wire passage	1.00 [0.46 to 2.15] (*P* = 1.00)	N/I
Degree of calcification	1.06 [0.78 to 1.43] (*P* = 0.67)	N/I
Calcified nodule	1.07 [0.52 to 2.19] (*P* = 0.85)	N/I
Degree of dissection after DCB	1.06 [0.64 to 1.74] (*P* = 0.79)	N/I
Minimum lumen area (mm^2^)	0.95 [0.87 to 1.04] (*P* = 0.20)	N/I
Distal external elastic membrane area (mm^2^)	1.01 [0.98 to 1.04] (*P* = 0.58)	N/I
Distal lumen area (mm^2^)	0.98 [0.92 to 1.03] (*P* = 0.36)	N/I

Data are hazard ratios [95% confidence intervals] (*P* values)

*N/I* not included

**Fig. 2:**
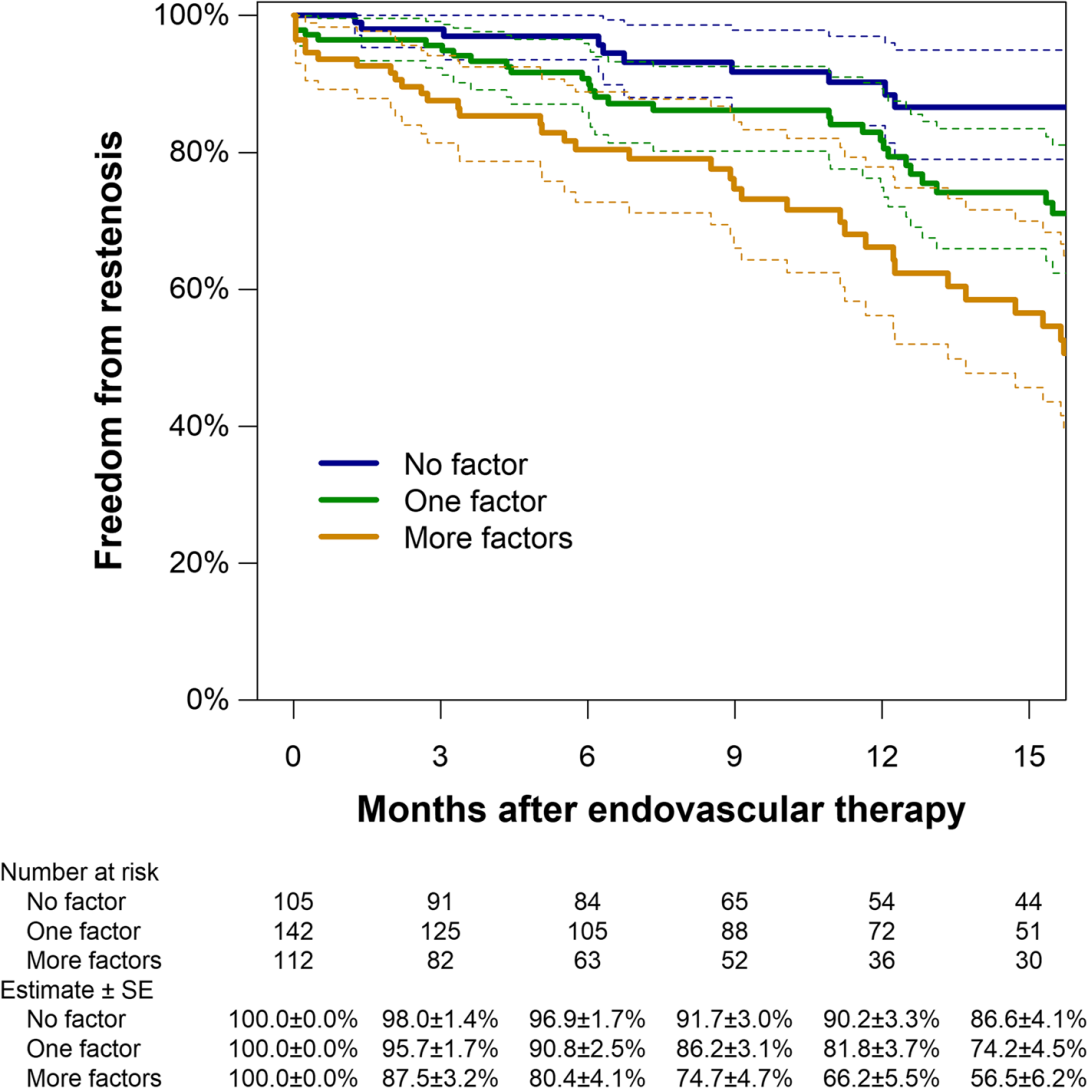
Kaplan Meier estimates of primary patency based on the accumulation of restenosis risk factors. Dotted lines indicate 95% confidence intervals. SE, standard error

In summary, 1) hemodialysis, 2) no use of DAPT, 3) CLTI, and 4) restenosis lesion were risks for 12-month restenosis. The accumulation of these risks was associated with a lower rate of 12-month primary patency; however, in the absence of these risks, a higher rate of primary patency was observed (no risk factor: 90.2%, one risk factor: 81.8%, and two or more factors: 66.2%, respectively, Fig. 2).

## Discussion

The present study demonstrated the clinical effectiveness of DCB treatment for patients with FP-CTO diseases. Multivariate analysis demonstrated three independent risk factors: 1) hemodialysis, 2) CLTI, and 3) restenosis lesion. The administration of DAPT was a significant protective factor. To the best of our knowledge, this is the largest population studied regarding the relationship between DCB and FP-CTO in real-world target populations. Compared with previous studies, we report a favorable rate of 12-month primary patency (79.8%) and acceptable bailout stent rate (8.9%); therefore, DCB treatment is an acceptable strategy for FP-CTO (AbuRahma et al. 2019; Tepe et al. 2019; Liistro et al. 2019).

Successful DCB treatment requires the achievement of two conflicting factors: 1) adequate luminal gain and less severe dissection. To establish these contradictory factors, the presence of CTO is very challenging. As reported in a previous study, the presence of CTO is significantly associated with the incidence of severe dissection (Fujihara et al. 2017). A large volume of plaques in the CTO may lead to large dissections and significant recoil after balloon angioplasty, causing target lesion failure (or requiring bailout stents). Recent studies have reported acceptable results of DCB treatment for complex FP lesions; however, they had a high rate (21.0%–46.5%) of bailout stent (Tepe et al. 2019; Liistro et al. 2019; Bausback et al. 2019). Currently, there is no high-level evidence regarding the effectiveness of the “leaving nothing behind” strategy for patients with complex FP lesions. A previous single-center study reported outstanding 12-month primary patency (92.7%) of IVUS-guided DCB treatment for FP-CTO without bailout stent (Hayakawa et al. 2022a, 2022b). They performed all procedures using an IVUS-guided intraluminal approach, and DCB size was decided based on the IVUS findings. Furthermore, only cases with sufficient results regarding lesion preparation (without residual stenosis [> 50%] and severe dissection [NHLBI grade D or higher]) were enrolled in the DCB cohort. Although the level of evidence was insufficient, their important findings suggested that precise procedures and judgment may improve the clinical outcomes of DCB treatment for complex FP lesions. Furthermore, this might also reduce the incidence of bailout stent, maximizing the benefit of the “leaving nothing behind” strategy. In our multicenter study, lesion length (21.9 cm), occlusion length (14.5 cm), and PACCS grade 3 or 4 (33.1%), were more severe than in the IN.PACT global CTO imaging cohort (lesion length: 22.8 cm, CTO length: 11.9 cm, severe calcification: 3.2%). Despite the lesion severity, the bailout stent rate was notably lower (8.9%) than that of the IN.PACT global study (46.5%) (Tepe et al. 2019). The reason for this is unclear; however, we suspect that the high rate of IVUS-guided procedures (77.2%) avoided the need for subintimal crossing and selection of an appropriate device size. As a result, favorable primary patency and lower bailout stent rates were observed. The first randomized controlled trial (RCT) of IVUS-guided FP EVT (mainly DCB treatment) demonstrated the clinical benefit of IVUS (Allan et al. 2022). In future clinical trials, it will be necessary to clarify 1) what IVUS changes in the procedure and 2) how it has affected the outcomes.

This study demonstrated that 1) hemodialysis, 2) CLTI, and 3) restenosis lesions were independent risk factors and that the use of DAPT was a protective factor for 12-month restenosis after DCB treatment for FP-CTO. The clustering of these risk factors was associated with a lower primary patency rate (66.2%). However, when none of these risk factors were present, an outstanding primary patency rate (90.2%) was noted. This might be beneficial in daily clinical practice because it can easily predict the lesion prognosis and stratify the risk, resulting in a better strategy selection. The risk factors identified in our study overlapped with the predictors of restenosis after fluoropolymer-based drug-eluting stent (FP-DES) implantation. Iida et al. reported hemodialysis, CLTI, and history of revascularization were risk factors for restenosis in FP-DES. A smaller reference vessel diameter, CTO, and spot stenting were also risk factors for restenosis (Iida et al. 2022). Hemodialysis, CLTI, and restenosis lesion might be associated with lesion severity, and therefore, might negatively influence durability after DCB treatment.

In this study, the administration of DAPT was demonstrated to be a protective factor for restenosis. It is well known that cilostazol prevents neointimal hyperplasia, resulting in a reduced restenosis rate after EVT for FP lesions (Soga et al. 2018). It is unclear how DAPT reduces restenosis after DCB treatment; however, DAPT might reduce the incidence of thrombosis-related target lesion failure, resulting in a reduction of restenosis. In this study, the effects of DAPT between restenosis and re-occlusion were not evaluated separately. Data on DAPT after EVT are scarce, and it is not clear whether longer DAPT provides more benefit. A previous report showed that DAPT was associated with prolonged survival for patients with critical limb ischemia who underwent arterial revascularization; however, no benefit was shown in patients with claudication (Soden et al. 2016). An RCT of the efficacy of DAPT (aspirin plus clopidogrel) reported reduced peri-interventional platelet activation and a lower revascularization rate than aspirin plus placebo (Tepe et al. 2012). Our study suggests DCB treatment for FP-CTO is reasonable for patients without CLTI, hemodialysis, or a history of target lesion EVT. Furthermore, if the patient has sufficient tolerance to DAPT (equal to non-high bleeding risk), the prolongation of DAPT should be considered. However, the current study did not examine the duration, dose, or regimen of DAPT or platelet reactivity. Further investigation is needed in the future.

In this study, previously reported risk factors for restenosis, such as age, sex, vessel diameter, lesion length, severe calcification, and dissection were not revealed as risks for restenosis. Furthermore, angioplasty with DCB was performed for cases with successful lesion preparation; therefore, DCB may be effective even for those with long lesions, small vessels ≤4 mm (Hiramori et al. 2017) or severe calcification if successful lesion preparation (sufficient luminal gain and less dissection) can be obtained. In this study, previously reported risk factors did not have an elevated risk for restenosis possibly because of the unclear consensus on the definition of successful lesion preparation, which differed between the current study and previous studies. Therefore, better selection of the lesions might lead to better results where the IVUS has a positive impact on the selection process (Hayakawa et al. 2022a, 2022b). Achievement of sufficient luminal gain and less severe dissection are essential. Regarding the definition of “sufficient” luminal gain, Horie et al. proposed a cutoff value of postprocedural IVUS-evaluated minimum luminal area (MLA) of 12.7 mm^2^ (Horie et al. 2022). Angiographic evaluation is the gold standard for the evaluation of dissection. A previous study showed a significant relationship between non-stented moderate-to-severe (angiographic) dissections after DCB treatment and the incidence of major adverse limb events (Giannopoulos et al. 2021). We evaluated dissections mainly by the NHLBI classification. Only 16% of the subjects had a grade C or more dissection. Therefore, it was difficult to evaluate the dissection pattern and durability. Kozuki et al. reported the presence of IVUS-detected severe dissection (dissection angle > 63°) was significantly associated with 12-month restenosis (Kozuki et al. 2021). In this study, 77.2% of subjects received IVUS-guided DCB treatment, possibly resulting in favorable patency without the need for bailout stent. However, over 20% of patients still received angiographic-guided treatment. Furthermore, none of the IVUS parameters was significantly associated with factors of restenosis in the current study. However, there were many cases with missing data of IVUS parameters. Therefore, we could not adequately evaluate the association between clinical outcomes and IVUS parameters (such as post-procedural MLA and dissection angle). DCB and IVUS together might help us to achieve better clinical results in connection with the strategy of DCB in cases with longer, smaller, or heavily calcified vessels, although further studies are required. In addition, the size and type of DCB was not an independent factor for restenosis in the present study and the type and size of balloon used to achieve optimal lesion preparation were not examined. Therefore, these issues require further investigation.

### Limitations

This study had several limitations. The present study was a retrospective, nonrandomized study with a small sample size; therefore, the evidence level is not high. In particular, the small sample size may be an important limitation with respect to the extraction of clinical events. In addition, patients with previous radiotherapy treatment or who had surgical femoropopliteal treatment could not be excluded in this study, so it is not known to what extent these patients were included. All clinical events were evaluated on-site and there was no independent clinical events committee. Furthermore, the lack of uniformity in follow-up modalities may have reduced the accuracy in terms of restenosis assessment. The application of DCB treatment was selected based on each operator’s decision without following any pre-established protocol. There may have been selection bias. The follow-up duration might have been too short for a thorough evaluation of the clinical outcome and complications. Moreover, the current study evaluated restenosis and re-occlusion but did not examine the details of complications such as aneurysmal degeneration after the DCB treatment. In the future, a well-designed, large-scale prospective study (with an independent event committee) will be required for equivalent evaluation. Because this was a single arm study of DCB, future studies should compare the results with other revascularization treatments.

## Conclusions

The 12-month clinical outcome of DCB treatment for FP-CTO demonstrated the effectiveness of DCB in real-world clinical practice. Independent risk factors for restenosis were hemodialysis, CLTI, and restenosis lesion. Therefore, the use of DAPT may reduce the risk of restenosis.

## Data Availability

The datasets used and/or analyzed during the current study are available from the corresponding author on reasonable request.

## Abbreviations

DCB: Drug-coated balloon
FP: Femoropopliteal
CTO: Chronic total occlusive lesions
CD-TLR: Clinically-driven target lesion revascularization
CLTI: Chronic limb-threatening ischemia
DAPT: Dual antiplatelet therapy
PAD: Peripheral artery disease
EVT: Endovascular therapy
NHLBI: The National Heart, Lung and Blood Institute
PACSS: Peripheral Arterial Calcium Scoring System
DUS: Duplex ultrasound
CTA: Computed tomography angiography
Cis: Confidence intervals
IVUS: Intravascular ultrasound
RCT: Randomized controlled trial
FP-DES: Fluoropolymer-based drug-eluting stent
MLA: Minimum luminal area
